# Variants in the Regulatory Region of *WNT5A* Reduced Risk of Cardiac Conotruncal Malformations in the Chinese Population

**DOI:** 10.1038/srep13120

**Published:** 2015-08-17

**Authors:** Peiqiang Li, Haijie Li, Yufang Zheng, Bin Qiao, Wenyuan Duan, Lijuan Huang, Weiqi Liu, Hongyan Wang

**Affiliations:** 1 The State Key Laboratory of Genetic Engineering, Ministry of Education (MOE) Key Laboratory of Contemporary Anthropology, Collaborative Innovation Center of Genetics and Development, School of Life Sciences, Fudan University, Shanghai 200438, China; 2 Institute of Cardiovascular Disease, General Hospital of Jinan Military Region, Jinan 250022, China; 3 Institute of Genetics, School of Basic Medical Sciences, Lanzhou University, Lanzhou City 730000, Gansu Province, China; 4 Children’s Hospital Shanghai, Fudan University, 399 Wanyuan Road, Shanghai 201102, China; 5 The Institutes of Biomedical Sciences, Fudan University, 138 Yixueyuan Road, Shanghai 200032, China

## Abstract

WNT5A is one of the most highly investigated non-canonical Wnt ligands and is involved in the embryonic heart development, especially in formation of the cardiac conotruncal region by regulating the migration and differentiation of cardiac neural crest (CNC) and second heart field (SHF) cells. No study to date has comprehensively characterized the *WNT5A* regulatory variants in patients with congenital heart malformations (CHMs). The association between regulatory variants of the *WNT5A* gene and CHMs was examined in case-control association study in 1,210 CHMs and 798 controls. Individuals carrying a homozygous genotype CC (rs524153) or GG (rs504849) had a similarly reduced risk of conotruncal malformations. The homozygous genotypes (CC for rs524153 and GG for rs504849) were associated with a lower *WNT5A* transcriptional level compared with the transcriptional level of those with wild-type genotypes. Further functional analysis revealed that an additional upstream single nucleotide polymorphisms (SNP) rs371954924 (–5244GCCA > CC) in a linkage disequilibrium (LD) block with the above genotyped SNPs decreased *WNT5A* expression through the attenuated binding affinity with the transcription factor SOX9. This is the first demonstration that genetic variants in the regulatory regions of *WNT5A* play a vital role in sporadic conotruncal malformations susceptibility through the changeable expression of the *WNT5A* gene.

Congenital heart malformations (CHMs) are structural problems that arise from the abnormal formation of the heart or its major blood vessels. CHMs account for nearly one-third of all major congenital anomalies and are the leading non-infectious cause of death during the first year of life^1^,^2^. CHMs affect nearly 1% of the population, and it is estimated that the population of adults with CHMs is growing at the rate of approximately 5% per year. The aetiology of CHMs is complex and involves both genetic and environmental factors^3^,^4^. Cardiogenesis is a very complex developmental process involving hundreds of genes that must be precisely regulated regarding both timing and spatial patterns of protein expression. During the last decade, more than 40 different genes have been implicated in non-syndromic CHMs^5^.

The Wnt signalling pathway includes canonical and non-canonical pathways and is one of the classical signalling pathways involved in cardiogenesis. The canonical Wnt signalling pathway works through β-catenin induced downstream activity, while the non-canonical Wnt signalling pathway is typically activated by a non-canonical Wnt ligand and acts independent of β-catenin transcriptional activity. WNT5A is one of the most extensively investigated non-canonical Wnt ligands involved in almost all aspects of non-canonical Wnt signalling. WNT5A regulates a variety of cellular functions, such as proliferation, differentiation, migration, adhesion and polarity^6^,^7^. The indispensable nature of Wnt5a during embryogenesis has been shown by the perinatal lethality of homozygous Wnt5a^−/−^ mice^8^. The Wnt5a protein is detected predominantly in structures undergoing extensive outgrowth, including the limbs, tail and facial structures^8^, and it controls limb elongation^9^ and cardiac neural crest (CNC) and second heart field (SHF) cell migration into cardiac outflow tract (OFT)^10^,^11^. Therefore, Wnt5a^−/−^ mutant mice exhibit caudal truncation with a shortened anterior-posterior axis, short fore- and hind-limbs with missing digits, and craniofacial malformation^8^. Notably, Wnt5a is highly expressed in mouse embryonic hearts, including pharyngeal mesoderm, SHF, OFT and compact myocardium of the ventricles and atria^12^,^13^,^14^. Wnt5a^−/−^ mice have OFT defects, including persistent truncus arteriosus (PTA), transposition of the great arteries (TGA), double outlet right ventricle (DORV) and interruption of the aortic arch (IAA)^10^. It has been known that both SHF and CNC progenitor cells migrate into OFT and participate the development of OFT and the remodelling of branchial arch arteries to form the great vessels of the heart^15^. Loss of Wnt5a reduces the growth of SHF and CNC progenitor cells in the developing heart and impairs the migration ability of CNC cells^10^,^13^,^14^.

Although the *WNT5A* gene plays an important role in the development of the heart and is closely related to many diseases, it has not yet been explored whether *WNT5A* is directly associated with CHMs. Only two missense mutations (p.Cys83Ser and p.Cys182Arg) in the *WNT5A* gene have been found in patients with Robinow syndrome, which is characterized by skeletal dysplasia and CHMs, especially right ventricular outflow obstruction^16^,^17^. Based on the results from the mouse model, we hypothesized that the genetic variants of *WNT5A* may be associated with human non-syndromic CHMs through functional impairment. In this study, we conducted an association study of the regulatory variants of *WNT5A* in 1210 Chinese patients with sporadic non-syndrome CHMs and 798 matched controls. We identified a functional SNP at –5244GCCA/CC (rs371954924) that reduced the risk of conotruncal defects by influencing *WNT5A* expression. These results confirmed that functional genetic variants of *WNT5A* contribute significantly to CHM susceptibility.

## Results

### *WNT5A* noncoding variants significantly reduce the risk of CNC-related CHM

**Figure 1:**
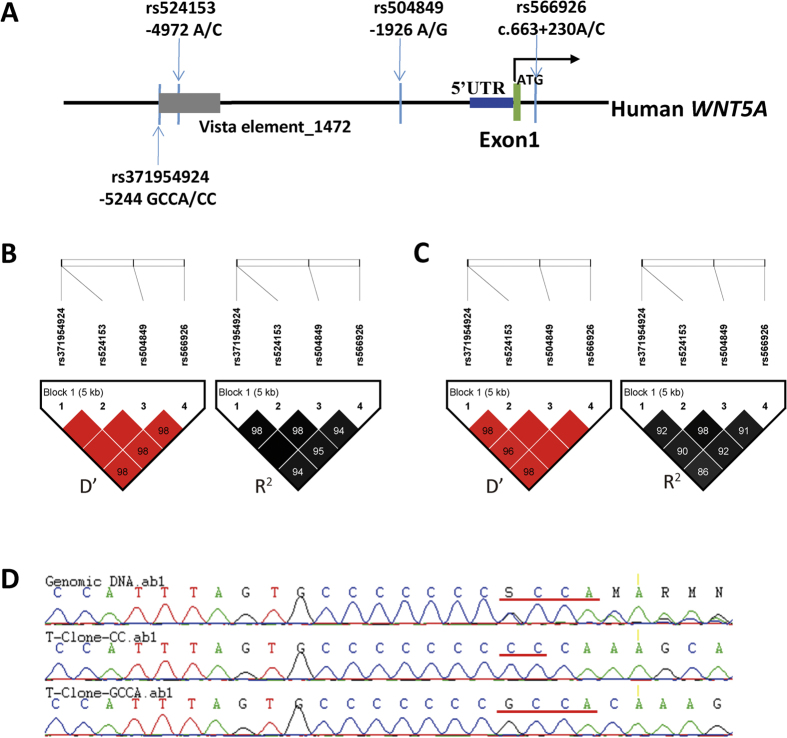
Four non-coding SNPs of *WNT5A* are in one haplotype. (**A**) The SNPs located in the promoter and enhancer-binding regions of *WNT5A* are indicated. According to Chinese population data from the 1000 Genome Project (**B**) and the genotyped results of 119 samples including 89 CHMs and 30 controls(**C**), the rs371954924 are in strong LD with other three SNPs (rs524153, rs504849 and rs566926) (D’ > 0.9 and R^2^ > 0.8). (**D**) By TA-clone re-sequencing of heterozygous samples, we confirmed the genotype of rs371954924 was GCCA/CC in our population.

To select the common SNPs with minor allele frequency (MAF) > 0.1 in the regulatory region of the *WNT5A* gene, we used Chinese Han data (CHB and CHS) from the 1000 Genomes Project. Two common SNPs, −1926 A/G (rs504849) and c.663 + 230 A/C (rs566926), were found in the promoter region (−2000 bp ~ +500 bp) of *WNT5A*. Meanwhile, according to the VISTA enhancer database, there is an enhancer element (element_1472) located in the upstream region −5247 ~ −3531 of the *WNT5A* gene, and there are two common SNPs, −4972 A/C (rs524153) and −5244 GCCA/CCC/CC/- (rs371954924), in this enhancer region as well (Fig. 1A). The haplotype analysis showed that the above four SNPs were in strong LD block (D’ > 0.8, R^2^ > 0.8) (Fig. 1B). Additionally, we observed that the genotype of rs371954924 was inconsistent between the 1000 Genomes Project database (C/-, Phase 3) and the reference SNP database (GCCA/CCC/CC/-, dbSNP 142). Therefore, we sequenced our samples by TA-clone re-sequencing of heterozygous samples. Our results showed that the genotype of rs371954924 was GCCA/CC in our population (Fig. 1D). To verify the rs371954924 (GCCA/CC) is within one LD with others three common SNPs located in the regulatory region of *WNT5A*, we randomly selected 119 samples, including 89 CHMs and 30 controls from our samples, and genotyped the four SNPs by Sanger sequencing. The results were analyzed with Haploview4.2 software and showed the rs371954924 (GCCA/CC) are in strong LD with other three SNPs (rs524153, rs504849 and rs566926) (D’ > 0.9 and R^2^ > 0.8) (Fig. 1C), which were also consistent with Chinese data from the 1000 Genome Project. It supported that the rs371954924 (CC) is present in the same haplotype with rs524153(C) and rs504849 (G) in our study population.

Since the four SNPs were strongly linked, then we genotyped two SNPs, one in the enhancer region (−4972 A/C) and one in the regulatory region (−1926 A/G) in 1,210 CHM patients and 798 controls by using SNaPshot (Supplemental Table 1). All genotype frequencies were calculated in accordance with the Hardy-Weinberg equilibrium among control subjects (*P* > 0.05). The observed MAF for the genotyped SNPs in the controls showed consistent with the published Chinese data from the 1000 Genomes Project (Table 1). Next, we conducted an association study of −4972 A/C and −1926 A/G between all CHM patients and control subjects, but there was no significant difference between the two groups either. However, different subtypes of CHMs have different cell origins and pathological causes, for example, functional abnormity of the progenitor cells from SHF and CNC contribute to the defects including DORV, TGA, PTA, conoventricular VSD, CoA and IAA, which are similar to the cardiac malformations found in conditional WNT5a^−/−^ mice^10^,^18^. Therefore, we re-analysed the two SNPs in subclassification groups of CHM. Interestingly, association was only observed in those conotruncal malformations. Individuals carrying the homozygous CC (−4972 A/C) or GG (−1926 A/G) genotypes had a reduced risk of conotruncal malformations than that of the AA + AC or AA + AG carriers when compared to the unrelated control population (−4972 A/C: OR = 0.56, 95% CI = 0.35–0.88, *P* = 0.017, recessive model AA + AC vs CC; −1926 A/G: OR = 0.55, 95% CI = 0.35–0.87, *P* = 0.015, recessive model AA + AG vs GG) (Table 2). No association was observed in other subtypes, such as septal defects (ASD, VSD) (Supplemental Table 3).

### The haplotype consisting of −5244 CC and −4972 C was associated with lower activity of *WNT5A*

**Figure 2:**
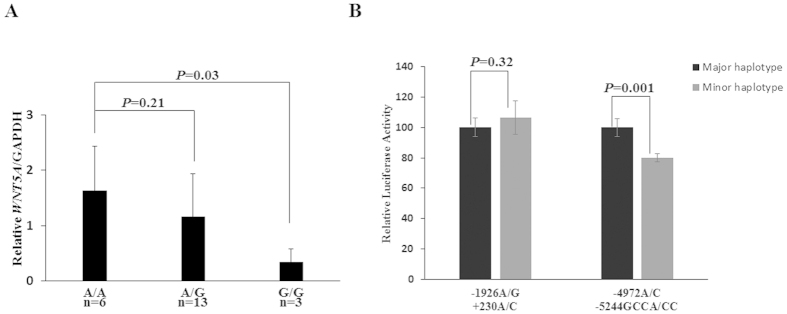
The *WNT5A* haplotype with −5244 CC exhibited lower transcriptional activity. (**A**) In individuals carrying different rs504849 genotypes, the mRNA level of *WNT5A* in the G/G genotype significant lower compared with that in the A/A genotype (*P* < 0.03). The actual values for each genotype group were as follows: A/A, 1.63 ± 0.80; A/G, 1.16 ± 0.77; G/G, 0.33 ± 0.25. All values have been normalized to the levels of *GAPDH* and represent the mean ± SD from 3 independent experiments. (**B**) Transcriptional levels of enhancer region (−4972 A/C + −5244GCCA/CC) and promoter region (−1926 A/G + 230 A/C) were respectively examined with luciferase assay in HEK293 cells. For the −4972 A/C + −5244GCCA/CC haplotypes, the luciferase expression level was significantly lower in the minor haplotype (CC-C haplotype) compared to that with the major haplotype (GCCA-A haplotype) (20% decreases). However, there were no significance differences in luciferase expression between major (A-A) and minor (G-C) haplotypes for −1926 A/G + 230 A/C haplotypes.

Because the associated polymorphisms were located in the 5′ upstream region of the *WNT5A* gene, it is very likely the transcription of *WNT5A* will be affected. To evaluate the transcriptional level of *WNT5A*, twenty-two human cardiovascular tissue samples were examined by qPCR and the results were analysed based on the genotypes. Our results showed that the samples with −4972 CC or −1926 A/G GG genotype displayed 80% reduced *WNT5A* mRNA expression compared to that in the samples with AA genotype (Fig. 2A).

To further investigate whether the reduction in *WNT5A* mRNA level was caused by the variants, two DNA fragments from the enhancer and promoter regions, −5551 to −4515 and −2237 to +672, were cloned into luciferase reporter plasmid, respectively. The former plasmid contains −5244 GCCA/CC and −4972 A/C variants, while the other one contains −1926 A/G and +230 A/C variants. Luciferase assays were performed to test the impact of different haplotype on transcriptional activity. The plasmid containing the haplotype (−5244CC + −4972C) displayed a significantly lower luciferase expression (20% reduction) than the reference haplotype (−5244GCCA + −4972A) in human HEK293 cells, while there was no significant differences observed for the haplotypes (−1926 A/G + 230 A/C) (Fig. 2B). These results from human tissues and cells confirmed that the haplotype (−5244CC + −4972C) located in enhancer region are associated with lower expression level of *WNT5A*.

### The variant **−**5244GCCA/CC attenuates the transcription factor SOX9 binding affinity

**Figure 3:**
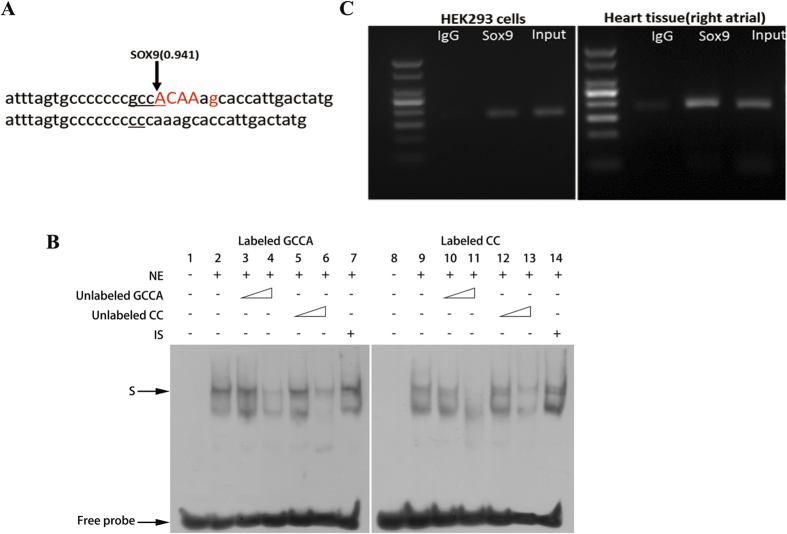
The *WNT5A* −5244 CC allele attenuates its binding affinity to SOX9. (**A**) MatInspector identified that the binding site of SOX9 is abolished by a −5244 GCCA to CC variation. The percentage matrix similarity score (in parentheses) is 0.941 (100% match to the matrix yields a maximum score of 1.00). Basepairs in capital characters indicate the core sequence. Basepairs in red characters show that the matrix exhibits a high conservation (Ci value > 60) at this position. (**B**) A competitive electrophoretic mobility shift assay on −5244GCCA/CC was performed using HEK293 nuclear extract. Lanes 1-7: biotin-labelled GCCA probe plus HEK293 nuclear proteins; Lanes 8-14: biotin-labeled CC probe plus HEK293 nuclear proteins. In the presence of nuclear extract, protein-DNA complexes are observed (lanes 2 and 9). 5- and 50-fold excesses of the unlabelled probes were used. The protein-DNA complexes were competed by increasing amounts of the unlabelled GCCA (lanes 3 and 4, and lanes 10 and 11) and CC probes (lanes 5 and 6, and lanes 12 and 13). The GCCA probes had a higher binding activity than the CC probes. (**C**) ChIP assays with HEK293 and a human tissue sample from right atrial. The presence of the SOX9-binding *WNT5A* enhancer was verified by PCR.

As the functional SNP is located in the enhancer region, it might affect the binding ability of transcription factors to the *WNT5A* gene. A bioinformatics analysis using software MatInspector predicted that the enhancer variant −5244GCCA/CC would influence the binding ability of the transcriptional factor sex-determining region Y-box 9 (SOX9); and the GCCA → CC variation could abolish the binding for SOX9 (Fig. 3A).

To experimentally confirm this interaction change, we conducted both electrophoretic mobility shift assay (EMSA) and chromatin immunoprecipitation (ChIP) assay. By using allele-specific probes, we examinated the DNA-protein interaction between the −5244GCCA/CC SNP and transcription factor in HEK293 nuclear extraction. The results showed that the binding affinity of the −5244GCCA allele probe was higher than that of the −5244CC allele probe (Fig. 3B). Then, we verified that SOX9 antibody could precipitate lysates from HEK293 cells and a cardiac tissue samples using ChIP assay, and the resulting PCR products which covered the −5244GCCA/CC indicated that the position of variant was occupied by SOX9. Our results showed that the WT (−5244GCCA) *WNT5A* enhancer can bind to SOX9, while the −5244CC allele has a lower binding affinity for transcription factor SOX9 (Fig. 3C). Such difference could cause a reduction on *WNT5A* transcription in people with CC genotype.

## Discussion

The development of embryonic conotruncal region is a transient cylinder vessel of myocardium connecting the embryonic ventricles to the aortic sac, and is a critical step in the establishment of the definitive circulatory system. Any events that disturbing the conotruncal development can impair its septation and/or remodelling, resulting in a spectrum of defects such as PTA, DORV, TGA and so on^19^,^20^. Normal conotruncal development required coordination between two population of extra-cardiac progenitor cells, SHF and neural crest cells. Their migration into OFT contribute to the septation and remodelling of the cardiac OFT. Evidence from animal model studies supports the fact that Wnt5a contributes to the conotruncal development. In mice, Wnt5a was expressed specifically in SHF progenitors, and it act in an auto- and/or paracrine fashion to regulated oriented cells migration into an epithelial-like sheet, which generated a pushing force to deploy SHF cells rostrally into the OFT^11^. Wnt5a was also highly expressed in the pharyngeal mesenchyme adjacent to the migrating neural crest cells and in the myocardial cell layer of the conotruncus to provide positional information for CNC cells^8^,^10^. Therefore, it is possible that changes of *WNT5A* expression were associated with risk of CHMs in humans. Our study showed for the first time that functional regulatory SNPs of the *WNT5A* gene are associated with sporadic CHMs. We discovered that the minor genotypes of the regulatory SNPs (GG in −1926 A/G and CC in −4972 A/C) of *WNT5A* significantly reduced the risk of conotruncal congenital malformations under a recessive model. However, such association did not exist in other sub-types of CHMs. It is likely that morphologically different subtypes of CHMs are related to different genetic backgrounds. Our study corroborated with other’s findings suggested that genetic variants associated with CHM exert high degree of phenotypic specificity^21^,^22^.

Our results also showed that the −5244 GCCA to CC variation in the enhancer region of *WNT5A* reduces its transcriptional level by influencing the SOX9-binding affinity. In mice, it has been shown that SOX9 is highly express not only in the neural crest-forming region but also in OFT region and to promote the proliferation and migration of neural crest cells and the development of OFT cardiac cushion^23^,^24^. Since Wnt5a is also found in OFT mesenchyme^13^, it is very likely SOX9 is important to regulate the enhancer activity of *WNT5A in situ* during embryonic cardiac development.

Notably, it was found here that lower *WNT5A* expression is associated with a reduced risk of conotruncal malformations. Our result correlates with the finding that *WNT5A* overexpressed mice have similar phenotypes to the knockout mice^25^. On the one hand, this indicates that the production of *WNT5A* needs to be precisely controlled. Too much WNT5A can destroy its gradient balance, resulting in a lack of positional cues which are required for directional elongation and migration. What’s more, overexpression of *WNT5A* could affect canonical Wnt signalling as well. Wnt1 and Wnt3a are the two canonical Wnt signalling ligands that are essential for the proliferation and migration of CNC and SHF cells to the OFT^15^,^26^,^27^. Both Wnt3a and Wnt5a bind to the same Fzd receptor on the cell surface^28^, a higher expression level of Wnt5a may interfere with Wnt3a in activating the canonical Wnt signalling.

The development of the heart involves in a large number of modifier genes, and genetic polymorphisms of modifier genes can buffer the effects of other major perturbations, such as rare mutations and damage from environmental teratogens^29^. Therefore, our study concludes that the −5244 GCCA/CC variant in *WNT5A* is an important modifying factor in reducing the risk of CHM. The transcription and expression of genes in cell lines and postnatal tissues may be not in conformity with the embryonic tissue; thus, the important role of this variant during cardiac development should be explored using stem cells and model animals in the future.

This is the first report describing common variants of the non-canonical Wnt gene *WNT5A* among human patients with sporadic CHMs. We demonstrated a significant association between the *WNT5A* −5244GCCA/CC (rs371954924) SNP and a reduced risk of conotruncal malformations in the Chinese Han population. Functional analysis demonstrated that the *WNT5A* −5244GCCA > CC variant down-regulates WNT5A expression through attenuated transcriptional SOX9 binding affinity. Identification of these loci provides a new perspective on the risk assessment for CHM and contributes to the understanding of *WNT5A* molecular mechanisms in regulating the proliferation and migration of SHF and CNC cells during cardiac outflow tract development.

## Materials and Methods

### Study Subjects

Blood samples from 1210 CHM patients (mean age 3.6 ± 3.5years, 51% male) were collected between 2008 and 2013 from the Cardiovascular Disease Institute of Jinan Military Command (Jinan, China). Routine clinical diagnoses were conducted, and patients with a variety of CHM were recruited. Detailed diagnosis information on the patients is shown in Supplemental Table 1. All of the CHM cases were classified according to the method described previously^30^. The 798 controls (mean age 10.3 ± 6.0years, 49% male) were ethnically gender-matched with unrelated healthy volunteers recruited from the same geographical area. CHM patients who manifested additional syndromes or who had a positive family history of CHM in a first-degree relative were excluded. For the quantitative RT-PCR assays, cardiovascular tissue samples were obtained from patients with CHM who had undergone a cardiac operation in the same hospital. The tissue from right atrial of TOF patient was applied to ChIP assay.

The present experiments on our participants were conducted in accordance with the Declaration of Helsinki. Protocols used in this work were reviewed and approved by the Ethics Committee of the School of Life Sciences, Fudan University prior to the commencement of the study. Written informed consent from the parents or guardians of the children was obtained.

### Genotyping of Candidate Single-Nucleotide Polymorphisms (SNPs)

Approximately 2 ml of peripheral blood was collected from each test subject. Genomic DNA of each test subject was isolated from peripheral blood using conventional reagents and was quantified using a NanoDrop2000 (Thermo Scientific).

Two common SNPs, rs524153 (−4972 A/C) and rs504849 (−1926 A/G) located in promoter and enhancer region of *WNT5A* gene (NM_003392) respectively, were selected as the candidate SNPs for genotyping. To confirm the correct genotype of rs371954924, heterozygous DNA fragment of three samples was cloned into pMD-18T vector (TaKaRa, Dalian, China), and ten clones of each sample were resequenced. The SNPs were genotyped with SNaPshot (ABI, Foster City, CA). Direct dye terminator sequencing of the PCR products was conducted with the ABI Prism BigDye according to the manufacturer’s instructions (ABI, Foster City, CA). The samples for sequencing and genotyping were run on an ABI3730 automated sequencer. Approximately 10% of the samples were examined by direct sequencing, and the results were also 100% concordant.

### Plasmid Construction and Site-Directed Mutagenesis

To construct the *WNT5A* reporter plasmid, we amplified two fragments, 2912 bp (−2237 ~ +672) and 1037 bp (−5551 ~ −4515), separately from genomic DNA with known homozygous genotypes after resequencing. The former fragment included −1926 A/C (rs504849) and c.663 + 230 A/G (rs566926), and the latter included −4972 A/C (rs524153) and −5244GCCA/CC (rs371954924). The two wild-type PCR products (−1926A + c.663 + 230A; −4972A + −5244GCCA) were subcloned into the pGL3 vector (Promega, Shanghai, China). Other two minor haplotypes (−1926C+ c.663 + 230G; −4972C + −5244CC) were generated by site-directed mutagenesis from a known haplotype, and the KOD was as a polymerase enzyme (Toyobo, Osaka, Japan). Following treatment with DpnI (NEB, UK), the products were used to transform E. coli DH5a to select mutant plasmid. All of the plasmids were validated by direct double-stranded sequencing. Supercoiled plasmids were purified on columns (Qiagen, Beijing, China) before transfection.

### Transcription Factor Binding Prediction

We used the *in silico* prediction software MatInspector^31^ to identify putative binding sites for predicted TFs around the specific target SNPs. TFs were then filtered for vertebrate specificity. For a given genomic region containing the target SNP, the software evaluates putative TFs binding sites in its directory and yields a matrix similarity score. We evaluated the impact of allelic changes contributed by the targeted SNPs. A perfect match of the matrix was given a score of 1.00, and a putative match was given a similarity score of >0.8. The consensus index (Ci) vector for the matrix represents the degree of conservation at each position within the matrix. A maximum Ci value of 100 is reached at a position with complete conservation of one nucleotide, whereas the minimum Ci value of 0 occurs only at a position with equal distribution of all four nucleotides and gaps. Red base pairs indicate that the matrix exhibits a high conservation at this position (Ci value > 60). Base pairs in capital characters denote the core sequence, which was defined as the highest conserved (usually 4) consecutive positions of the matrix.

### Cell Culture and Luciferase Assay

Human embryonic kidney 293 (HEK293) cells were grown in DMEM supplemented with 10% FBS. HEK293 cells were transfected (24 h after 1 × 10^5^ seeding in 24-well plate) with 500 ng of promoter haplotype-firefly luciferase reporter plasmid and 10 ng of the renilla luciferase expression plasmid pRL-TK (Promega, Shanghai, China) as an internal control per well using Lipofectamine2000 (Invitrogen, Shanghai, China). The firefly and renilla luciferase activities in cell lysates were measured 24 h after transfection using the Dual Luciferase reporter assay system (Promega, Shanghai, China). Three independent transfection experiments were performed, and each luciferase assay was performed in triplicate. Results were expressed as the mean ± SD. Statistical significance was calculated using the Student t-test, and statistical significance was established at *P* < 0.05.

### Electrophoretic Mobility Shift Assay (EMSA)

Nuclear proteins were extracted from HEK293 cells using NE-PER nuclear and cytoplasmic extraction reagents (Pierce, USA). Duplex oligonucleotide probes representing the −5244 GCCA or CC alleles (sequences listed in Supplemental Table 2) were labelled with biotin. The EMSAs were performed using a Light Shift Chemiluminescent EMSA kit (Pierce, USA) according to the manufacturer’s protocols. Briefly, 1 pmol of biotin-labelled duplex oligonucleotides bearing either −5244 GCCA or CC alleles was incubated with 8 μg of nuclear extracts for 20 min in 10 × binding buffer supplemented with 1 μg/μl poly (dI•dC). Unlabelled probes at 5- and 50-fold molar excesses, as indicated, were added to the reaction for competition. The reaction mixture was electrophoresed on a native 8% polyacrylamide gel and transferred to a positive nylon membrane. The biotin-labelled DNA was detected using stabilized streptavidin-horseradish peroxidase conjugate (Pierce, USA) and exposure to X-ray film.

### Chromatin Immunoprecipitation (ChIP) Assays

A ChIP assay was performed using the MAGnify ChIP Kit (Invitrogen, USA). HEK293 Cells (10 × 10^6^ cells) and heart tissue (50 mg) were cross-linked in 1% formaldehyde for 10 min at 37 °C and washed with ice-cold PBS, then resuspended in cell-lysis buffer. The DNA was fragmented to 200 ~ 500 bp using Bioruptor (Diogenode, Liege, Belgium). Immunoprecipitation of the sheared chromatin was performed using anti-SOX9 antibody (ab71762, Abcam) or nonspecific rabbit IgG (Santa Cruz Biotechnology, Santa Cruz, CA) incubated overnight at 4 °C. Final DNA extractions were PCR-amplified using the primer pairs (Supplemental Table 2) that defined a 112 base-pair(bp) product which covered the variant −5244GCCA/CC.

### Quantitative Real-Time PCR

Total RNA was extracted from human cardiovascular tissue samples using the miRNeasy Mini Kit (Qiagen, Beijing, China) and was converted to cDNA using random hexamers, oligo(dT) primers and Moloney murine leukaemia virus reverse transcriptase (TaKaRa, Dalian, China). *WNT5A* mRNA levels were measured with qPCR using the StepOnePlus system (ABI, Foster City, CA), with *GAPDH* as an internal reference gene. The reaction mixture contained 10 μM of each primer, 2 × SYBR Green PCR Master Mix (ABI, CA, USA) and 1 μl cDNA. The primers that were used are listed in Supplemental Table 2. Each reaction was performed in triplicate.

### Statistical analysis

The Hardy-Weinberg equilibrium (HWE) was tested using the χ2 test for the controls. Odds ratios (ORs) and 95% confidence intervals (CI) were calculated using unconditional logistic regression analysis with adjustment for sex. The analyses of the association between different haplotypes and CHM risk were performed using the SNPStats (http://bioinfo.iconcologia.net/snpstats/start.htm). We reconstructed haplotypes of the SNPs using Haploview version 4.2. All statistical tests were two-tailed with *P* < 0.05 set as the significance level.

## Additional Information

**How to cite this article**: Li, P. *et al.* Variants in the Regulatory Region of *WNT5A* Reduced Risk of Cardiac Conotruncal Malformations in the Chinese Population. *Sci. Rep.* **5**, 13120; doi: 10.1038/srep13120 (2015).

## Supplementary Material

Supplementary Information

## Figures and Tables

**Table 1:** Association of *WNT5A* rs524153 and rs504849 variants with CHM in all samples.

**SNPs**	**Genetic model**	**Pattern**	**Control**	**Case**	***P***-value*	**MAF**			**OR (95% CI)**
						**Control**	**Case**	**Database**#	
rs524153	Codominant	AA/AC/CC	336/351/111	498/569/143	0.25	0.36	0.35	0.37	NA
	Dominant	AA/AC + CC	336/462	498/712	0.69				1.04 (0.87–1.24)
	Recessive	AA + AC/CC	687/111	1067/143	0.16				0.83 (0.63–1.08)
rs504849	Codominant	AA/AG/GG	339/347/112	508/554/148	0.5	0.36	0.35	0.37	NA
	Dominant	AA/AG + GG	339/459	508/702	0.75				1.03 (0.86–1.24)
	Recessive	AA + AG/GG	686/112	1062/148	0.33				0.88 (0.67–1.14)

^*^Adjusted by sex; OR = odds ratio; CI = confidence interval; NA = not available.

^#^MAF = minor allele frequency from data of Han Chinese in the 1000 Genome Project.

**Table 2:** Association of *WNT5A* rs524153 and rs504849 variants with conotruncal malformations.

**SNPs**	**Genetic model**	**Pattern**	**Control**	**Case**	***P***-value*	***P*****c-value**#	**OR (95% CI)**
rs524153	Codominant	AA/AC/CC	336/351/111	138/133/25	0.026	0.052	NA
	Dominant	AA/AC + CC	336/462	138/158	0.16	0.32	0.82 (0.63–1.08)
	Recessive	AA + AC/CC	687/111	271/25	0.0086	0.017	0.56 (0.35–0.88)
rs504849	Codominant	AA/AG/GG	339/347/112	139/132/25	0.024	0.048	NA
	Dominant	AA/AG + GG	339/459	139/157	0.15	0.3	0.82 (0.63–1.08)
	Recessive	AA + AG/GG	686/112	271/25	0.0076	0.015	0.55 (0.35–0.87)

^*^Adjusted by sex.

^#^corrected P value (after Bonferroni multiple adjustment); OR = odds ratio; CI = confidence interval; NA = not available.
