# Supplementary material for: Variants in the Regulatory Region of WNT5A Reduced Risk of Cardiac Conotruncal Malformations in the Chinese Population
Source: Sci Rep. 2015 Aug 17;5:13120. doi: 10.1038/srep13120 (PMC4538571; doi:10.1038/srep13120)
Supplement: Supplementary Information [file srep13120-s1.doc]

**Variants in the Regulatory Region of *WNT5A* Reduced Risk of Cardiac Conotruncal Malformations in the Chinese Population**

Peiqiang Li^1,3^, Haijie Li^2^, Yufang Zheng^1^, Bin Qiao^2^, Wenyuan Duan^2^, Lijuan Huang^1^, Weiqi Liu^1^, Hongyan Wang^1,4,5*^

**Supplemental Table 1. Phenotypes of screened population with congenital heart defects**

| **Cardiac diagnosis** | **Number** |
| --- | --- |
| Atrial Septal Defect (ASD) | 87(7.2%) |
| Ventricular Septal Defect (VSD) | 409(33.8%) |
| ASD/VSD | 91(7.5%) |
| Atrioventricular Septal Defect (AVSD) | 42(3.5%) |
| Trilogy of Fallot (TOF) | 124(10.2%) |
| conoventricular VSD | 29(2.4%) |
| Double Outlet Right Ventricle(DORV) | 66(5.5%) |
| dextro-Transposition of the Great Arteries (d-TGA) | 41(3.4%) |
| Persistent Truncus Arteriosus(PTA) | 5(0.4%) |
| Coarctation of the Aorta (CoA) | 28(2.3%) |
| Interrupted Aortic Arch (IAA) | 3(0.2%) |
| Aortic Stenosis (AS) | 22(1.8%) |
| Pulmonic Valve Stenosis (PVS) | 79(6.5%) |
| Pulmonary Atresia (PA) | 28(2.3%) |
| Tricuspid Atresia (TA) | 3(0.2%) |
| Ebstein's anomaly | 8(0.7%) |
| Patent Ductus Arteriosus (PDA) | 86(7.1%) |
| Heterotaxy | 13(1.1%) |
| Anomalous Pulmonary Venous Return (APVR) | 28(2.3%) |
| Complex defect | 18(1.5%) |
| All | 1210(100%) |

**Supplemental Table 2. DNA sequence of all used primer pairs**

| **Primer Name** | **Sequence (5’-3’)** | **Purpose** |
| --- | --- | --- |
| rs524153 F | ATAATCACAGTAGCTGCCATTC | PCR/Sequence |
| rs524153 R | TGGAGCCCTAAAGAAGACAA | PCR/Sequence |
| rs504849 F | TGCTAAGCAGGAGCATCTGTC | PCR/Sequence |
| rs504849 R | GGGACCTCCTCATTCTTACCTCT | PCR/Sequence |
| rs566926 F | CCTCGCCATGAAGGTAGGTG | PCR/Sequence |
| rs566926 R | TGTACGCCGCTCTGGAGTAGTT | PCR/Sequence |
| rs524153 | ttttttttttttttttttACCGTCAAAGTAATACCATC | Genotype |
| rs504849 | ttttttttttttttttGTGAGCGCCTAACCCTATCC | Genotype |
| Plasmid-1F | CGGGGTACCTGTGAGGCACTGTTTGTGGG | Construct |
| Plasmid-1R | CCGCTCGAGGGCGGCTAATAATGCTAATAACG | Construct |
| Plasmid-2F | CGGGGTACCGGGACCCAGGAACAGACATT | Construct |
| Plasmid-2R | CCCAAGCTTCCCAACCGGATTATTCACAA | Construct |
| -5244-GCCA-F | GTGCCCCCCCGCCACAAAGCACCAT | EMSA |
| -5244-GCCA-R | ATGGTGCTTTGTGGCGGGGGGGCAC | EMSA |
| -5244-CC-F | GTGCCCCCCCCCCCAAAGCACCAT | EMSA |
| -5244-CC-R | ATGGTGCTTTGGGGGGGGGGGCAC | EMSA |
| CHIP-F | CCCTAAAATGAAACAAAATTAAAGC | CHIP |
| CHIP-R | CACTTATGTGTTATGTGTTATATGT | CHIP |
| *WNT5A*-qPCR-F | ATTCTTGGTGGTCGCTAGG | RT-qPCR |
| *WNT5A*-qPCR-R | TCCTTGAGAAAGTCCTGCC | RT-qPCR |
| GAPDH-qPCR-F | GAAACTGTGGCGTGATGGC | RT-qPCR |
| GAPDH-qPCR-R | CACCACTGACACGTTGGCAG | RT-qPCR |

**Supplemental Table 3. Association of *WNT5A*** rs524153 and rs504849 variants with septal defects

| **SNPs** | **Genetic model** | **Pattern** | **Control** | **Case** | ***P*-value** | ***P*c-value^#^** | **OR (95% CI)** |
| --- | --- | --- | --- | --- | --- | --- | --- |
| **rs524153** | Codominant | AA/AC/CC | 336/351/111 | 215/299/73 | 0.037 | 0.074 | NA |
|  | Dominant | AA/AC+CC | 336/462 | 215/372 | 0.039 | 0.078 | 1.33 (1.06-1.68) |
|  | Recessive | AA+AC/CC | 687/111 | 514//73 | 0.42 | 0.84 | 1.03 (0.73-1.45) |
| **rs504849** | Codominant | AA/AG/GG | 339/347/112 | 216/295/76 | 0.041 | 0.082 | NA |
|  | Dominant | AA/AG+GG | 339/459 | 216/371 | 0.033 | 0.066 | 1.33(1.06-1.68) |
|  | Recessive | AA+AG/GG | 686/112 | 511/76 | 0.56 | 1.12 | 1.07 (0.76-1.49) |

*Adjusted by sex;^#^corrected P value (after Bonferroni multiple adjustment); OR = odds ratio; CI = confidence interval;

NA = not available
